# Antifungal Effect of Nanoparticles against COVID-19 Linked Black Fungus: A Perspective on Biomedical Applications

**DOI:** 10.3390/ijms232012526

**Published:** 2022-10-19

**Authors:** Sangiliyandi Gurunathan, Ah Reum Lee, Jin Hoi Kim

**Affiliations:** 1 Department of Stem Cell and Regenerative Biotechnology, Konkuk University, Seoul 05029, Korea; 2 CHA Advanced Research Institute, CHA Medical Center, 335 Pangyo-ro, Bundang-gu, Seongnam-si 13488, Korea

**Keywords:** COVID-19, mucormycosis, silver nanoparticles, gold nanoparticles, zinc oxide nanoparticles, copper oxide nanoparticles, magnetic and iron nanoparticles

## Abstract

Severe acute respiratory syndrome coronavirus 2 (SARS-CoV-2) is a highly transmissible and pathogenic coronavirus that has caused a ‘coronavirus disease 2019’ (COVID-19) pandemic in multiple waves, which threatens human health and public safety. During this pandemic, some patients with COVID-19 acquired secondary infections, such as mucormycosis, also known as black fungus disease. Mucormycosis is a serious, acute, and deadly fungal infection caused by Mucorales-related fungal species, and it spreads rapidly. Hence, prompt diagnosis and treatment are necessary to avoid high mortality and morbidity rates. Major risk factors for this disease include uncontrolled diabetes mellitus and immunosuppression that can also facilitate increases in mucormycosis infections. The extensive use of steroids to prevent the worsening of COVID-19 can lead to black fungus infection. Generally, antifungal agents dedicated to medical applications must be biocompatible, non-toxic, easily soluble, efficient, and hypoallergenic. They should also provide long-term protection against fungal growth. COVID-19-related black fungus infection causes a severe increase in fatalities. Therefore, there is a strong need for the development of novel and efficient antimicrobial agents. Recently, nanoparticle-containing products available in the market have been used as antimicrobial agents to prevent bacterial growth, but little is known about their efficacy with respect to preventing fungal growth, especially black fungus. The present review focuses on the effect of various types of metal nanoparticles, specifically those containing silver, zinc oxide, gold, copper, titanium, magnetic, iron, and carbon, on the growth of various types of fungi. We particularly focused on how these nanoparticles can impact the growth of black fungus. We also discussed black fungus co-infection in the context of the global COVID-19 outbreak, and management and guidelines to help control COVID-19-associated black fungus infection. Finally, this review aimed to elucidate the relationship between COVID-19 and mucormycosis.

## 1. Introduction

The spread of severe acute respiratory syndrome coronavirus 2 (SARS-CoV-2), the virus-causing coronavirus disease 2019 (COVID-19), has led to a fourth wave of infection and immense mortality. COVID-19 can have sub-acute and prolonged long-term symptoms that can eventually damage multiple organ systems [1]. According to the World Health Organization (WHO), as of 15 October 2022, there have been 620,878,405 confirmed cases of COVID-19, including 6,543,138 deaths. As of 15 October, a total of 12,782,955,639 vaccine doses have been administered. Mucormycosis is an aggressive, severe, and rare fungal infection that can affect the patients’ pre- and post-COVID-19. Recently, post-COVID-19 infections caused by members of the order Mucorales have gained importance due to their higher incidence in immunocompromised patients with mucormycosis. They include some of the most common molds causing infections and ranked third for causing invasive infections in organ transplant and malignant tumor cases [2]. Based on the infection site, mucormycosis is clinically classified as rhino-orbital, cutaneous, pulmonary, disseminated, or gastrointestinal. Other forms can cause renal infections, osteomyelitis, endocarditis, and peritonitis and are invasive and aggressive. They can grow within blood vessels and cause thrombosis and tissue necrosis. This ability also enables hematogenous fungal dissemination [2,3].

Patients with COVID-19 are extremely prone to fungal and bacterial infections. Aspergillosis, invasive candidiasis, and mucormycosis are the most common fungal infections [4,5,6,7,8,9]. The most significant risk factors for the development of mucormycosis are diabetes mellitus, prolonged steroid use, hematological malignancy, post-stem cell transplant status, prolonged neutropenia, and impaired cell-mediated immunity [10,11]. Mucormycosis frequently affects the sinus, brain, and respiratory systems. A study on fungal cultures from 99 patients with COVID-19 found five (5%, 5/99) cases of fungal infections. These included one case of infection with *Aspergillus flavus*, one case with *Candida glabrata*, and three cases with *Candida albicans* [12]. Another study reported that patients with COVID-19 showed a higher percentage (8–15%) of secondary infections [13,14]. Koehler et al. [7]. reported that COVID-19-associated invasive pulmonary aspergillosis was found in 5 (26.3%) out of 19 consecutive critically ill patients with moderate-to-severe acute respiratory distress syndrome. A study from the Netherlands reported that among patients with COVID-19 who stayed in the ICU, 5 out of 31 patients were infected with *Aspergillus fumigatus*, and older severely ill patients were co-infected with *A. flavus*, as determined by tracheal aspirate culture [15,16]. Patients with COVID-19 are more susceptible to mucormycosis or other fungal co-infections due to the overexpression of inflammatory cytokines and an impaired cell-mediated immune response, with decreased CD4^+^ and CD8^+^ T cell counts. Soliman et al. [17]. reported that the toxin produced by Mucorales plays a significant role in virulence. As a result produced polyclonal antibodies against this toxin lower its ability to damage human cells in vitro, and can prevent hypovolemic shock, organ necrosis, and death in mice with mucormycosis. 

The most important factors for effective antifungal therapy are early diagnosis and treatment. Moreover, surgical removal of all infected tissues and adjunctive therapies are the most effective ways to eradicate mucormycosis. For example, amphotericin B is the preferred antifungal drug for the treatment of mucormycosis. Further treatment may include triazoles, such as posaconazole, isavuconazole, and voriconazole. Recently, nanotechnology-driven innovations have provided a solution to overcome the problem of drug resistance and hence can have a profound influence on the improvement of human health. Nanotechnology-based antimicrobial agents have shown enhanced durability, performance, strength, flexibility, and unique physicochemical properties. Different types of nanoparticles have shown excellent antifungal activities and are considered a good alternative for controlling various types of fungi [18]. Specifically, metal nanoparticles can play a significant role due to their strong antifungal properties [19]. 

Over the past several years, the number of fungal infections has continuously increased. Over 70% of reported cases are caused by *Candida* spp., especially in patients with immune disorders and other serious diseases, as well as those hospitalized with serious underlying diseases [20]. Members of *Candida* are the most common fungal pathogens, causing diseases ranging from superficial (oral and vaginal) to systemic (such as peritonitis and meningitis candidiasis) [21]. Although a large number and diverse group of antibacterial drugs are currently available, the number of active substances for the treatment of pathogenic fungi is limited. Recent studies suggest that an increasing number of *Candida* infections are resistant to currently available classes of antifungal agents [22]. Hence, studying new therapeutic approaches is required for the development of novel antifungal strategies. The increased resistance of fungal pathogens to current treatments and the lack of alternative and effective treatment options are some of the greatest challenges in current medicine. Various types of therapeutic modalities, including combined drug therapy, chemical structure modifications, and the use of drug transporters, have been employed to overcome pathogenic strain resistance [23,24]. For example, several studies have found that nanoparticles can inhibit the function of efflux drug transporters, promote apoptosis of fungal cells, and reduce the formation of biofilm [25]. One study suggested that iron oxide-based nanosystems could inactivate catalase Cat-1 and thus provide a new strategy for overcoming fungal resistance. Recently, the development of nanotechnology in various applications has been rapid and immense. Nanotechnology is a new arena that focuses on particles with sizes ranging from approximately 1 to 100 nm. Nanoparticles (NPs) are widely used in nanomedicine against bacterial and fungal infections, and there is a low probability that pathogens can develop resistance to them. NPs are considered suitable candidates for transporting drugs to their targets [26,27,28]. Several types of metal and metal oxide NPs, such as silver (Ag), silver oxide (Ag_2_O), titanium dioxide (TiO_2_), zinc oxide (ZnO), gold (Au), calcium oxide (CaO), silica (Si), copper oxide (CuO), and magnesium oxide (MgO), are known to show antimicrobial activity [29]. The antimicrobial properties of nanoparticles depend on their composition, surface modification, intrinsic properties, and the type of microorganism [30]. Recently, Mal’tseva et al., reported that selenium-containing compounds such as sodium selenite, methylseleninic acid, selenomethionine, and methylselenocysteine, as well as selenoproteins and selenium nanoparticles, are involved in the regulation of defense mechanisms against various viral infections, including coronavirus infection (COVID-19) [31].

Previous studies have demonstrated that when NPs were combined with either antimicrobial peptides or antibiotics, they exhibited potential antimicrobial activity at low concentrations [32,33]. The interaction between the fungal cells and the metal is necessary for fungal resistance and homeostasis [34,35,36]. Fungi are exploited for the synthesis of nanoparticles with antimicrobial properties because they have high levels of natural metal resistance, can be economically grown, and are capable of mass production [37,38,39]. Silver is a transition metal that has properties in common with other transition metals. Silver is considered to be an effective antifungal agent for the eradication of pathogens. Silver has a positive effect on intestinal microflora, aflatoxins, and mycotoxin absorption in farm animals, and it is used in the food industry for packaging because of its antimicrobial properties [40]. Generally, Ag^+^ has a high affinity for sulfhydryl (thiol) and phosphate groups in enzymes required for cell wall synthesis in bacteria and fungi. In addition, it can interfere with the electron transport chain and the generation of energy. Ag^+^ also inhibits DNA replication and the respiratory chain in bacteria and fungi and ultimately causes cell death by generating reactive oxygen species (ROS) [41,42,43]. Gold nanoparticles (AuNPs) are generally considered to be biocompatible, non-toxic, and safer than other inorganic nanoparticles. The redox nature of Au is beneficial in reducing the ROS levels produced during exposure to NPs [44,45]. Several studies have confirmed that AuNPs exhibit significant antimicrobial activity against various clinical strains of *Candida* [34,46].

Zinc oxide nanoparticles (ZnO NPs) are more active antimicrobial agents against various types of microbes than other NPs in the same group of elements. Because ZnO NPs have potential UV-absorbing properties and high catalytic efficiency, they have been widely used in cosmetic lotions and as successful UV blockers. They have also been used as semiconductors in microelectronic devices and for accelerating the degradation of water pollutants by photocatalytic activity [47]. ZnO NPs exhibited enhanced cytotoxicity by increasing the production of ROS, which leads to oxidative stress and eventual cell death [48,49]. ZnO NPs had significant inhibitory effects on the growth of the *C. albicans* fungus. These NPs inhibit the growth of *C. albicans* by directly contacting the fungal cell membrane and affecting its interaction with the cell wall which can prevent growth and lead to cell death [50]. The exposure of fungal cells to NPs reduces the effectiveness of their anti-oxidative mechanisms and oxidative enzymes, and also disrupts their osmotic balance and cellular integrity. Carbon-based nanoparticles, such as fullerenes, single-walled carbon nanotubes (SWCNTs), graphene oxide (GO), and reduced GO, also exhibit antimicrobial activity (Gurunathan et al., 2012) [51]. Carbon-based nanomaterials cause membrane damage in microorganisms due to oxidative stress. The interactions between microbial cells and carbon-based nanomaterials play a significant role in their antimicrobial activity. Carbon nanomaterials can come into direct contact with the cells, which causes cell death [52,53,54,55]. Copper is an important biocompatible element involved in various physiological processes and has been effectively used to eradicate microorganisms. 

Titanium dioxide (TiO_2_) is an inorganic NP that is non-toxic, antibacterial, and super lipophilic. It also promotes bacterial decomposition and protects against ultraviolet (UV) rays [56,57]. Because of these factors and its broad range of functions, TiO_2_ is among the most widely used NPs in industry and consumer applications. There are three forms of TiO_2_ crystal structures: anatase, rutile, and brookite [58]. TiO_2_ NPs exhibit potential antimicrobial activities as observed against *C. albicans* in the presence or absence of sunlight. Iron oxide NPs (IONPs) are considered safe and non-toxic and are widely used because of their magnetic and biochemical properties as well as their low cost of use in various medical applications. IONPs have been shown to have broad-spectrum antiviral [59], antiparasitic [60], bactericidal [61,62], antibiofilm, antifungal [63], and wound healing properties [64]. IONPs have attracted immense interest due to their remarkable physiochemical properties and have been utilized in a broad range of applications, including biomedicine, electronics, catalysis, water treatment, and magnetic and environmental remediation [65,66]. 

Several studies have reported the development of severe opportunistic infectious diseases, such as pneumonia, candidiasis, and pulmonary aspergillosis, in patients with COVID-19 [67]. Mucormycosis is an acute and invasive fungal infection caused by Mucorales-related fungal species, and it is significantly more likely to occur in immunocompromised patients. Mucormycosis infections are caused by ubiquitous opportunistic fungi belonging to the order Mucorales, family Mucoraceae, and they are commonly known as the black fungus [68,69]. The novel SARS-CoV-2 causes susceptibility to two different fungal infections, COVID-19-associated pulmonary aspergillosis (CAPA) and COVID-19-associated mucormycosis (CAM) [70]. One study found that more than 47,000 cases of CAM were reported in merely three months in India [71]. Patients with diabetes are at a higher risk of infection with COVID-19-linked black fungus because of their high levels of blood sugar. Researchers believe that high blood sugar levels make the body a more conducive environment for the growth of fungi. Another risk factor is the use of corticosteroids, which are strong immunosuppressants used to treat severe cases of COVID-19 [71,72]. The mortality rate was significantly increased in COVID-19 patients with black fungus infection. Among COVID-19-infected countries, the mortality rate of black fungus-infected patients was significantly higher in India [73]. The symptoms of mucormycosis, such as reddish swollen skin over the nose and around the eyes, have been observed. Other symptoms include vision abnormalities, eye swelling, ocular pain, facial edema, and shortness of breath [74]. Rapid diagnosis of mucormycosis can be performed using fluorescent brighteners. Furthermore, fungal culturing techniques allow identification at the genus and species levels. Quantitative PCR is a very sensitive and rapid method to diagnose mucormycosis in patients who cannot undergo a biopsy or in patients with hematological malignancies due to severe thrombocytopenia [75,76].

This review presents a wide-ranging and detailed analysis of the current progress and challenges in the application of metal nanoparticles for controlling COVID-19-associated black fungus infection. Particularly, we discuss the effects of nanoparticles on fungal infections and the use of various types of metal nanoparticles as potential antifungal agents against different types of fungi. Further, we discuss the pathogenesis of mucormycosis, and the effect of nanoparticles on black fungus, and also we discuss black fungus co-infection in the context of the global COVID-19 outbreak. Then, we discuss the management and guidelines to control COVID-19-related black fungus infection. Finally, we present our conclusions and possible directions for further research.

## 2. Silver Nanoparticles

Engineered silver nanoparticles (AgNPs) are some of the most common nanoproducts used against microorganisms [28,77,78,79]. The toxic nature of AgNPs depends on their size, composition, surface area, charge, redox potential, and concentration [80,81]. When the fungus *Arthroderma fulvum* strain HT77 was used to biologically synthesize AgNPs, the resulting nanoparticles exhibited antifungal activity against a variety of fungal pathogens, including *Candida* spp., *Aspergillus* spp., and *Fusarium* spp. Pd@Ag nanosheets (Pd@Ag NSs) also exhibited excellent inhibitory antifungal activity against common invasive fungal pathogens, such as those in the *Cryptococcus neoformans* species complex. This included fluconazole-resistant isolates. The anticryptococcal activity of the Pd@Ag NSs was significantly greater than that of fluconazole and similar to that of amphotericin B (AmB). Pd@Ag NSs exhibited fungicidal activity against *Cryptococcus* spp. by disrupting cell integrity, intracellular protein synthesis, and energy metabolism [28]. AgNPs were found to have potent antifungal activity against *Candida* by generating ROS. They also affect other cellular targets, resulting in alterations in membrane fluidity, cellular morphology, and ultrastructure t as well as ergosterol content [82]. AgNPs synthesized from crude bioactive metabolites of bionanofactories isolated from Lake Mariout exhibited antibacterial, antifungal, and antibiofilm activities. Silver/chitosan nanocomposites with an average size of 10 nm have the potential to inhibit the growth of *Penicillium expansum,* which is the causative agent of blue mold-contaminated dairy cattle feed [83]. AgNPs synthesized from aqueous pigeon dropping (PD) extract inhibited various types of microorganisms, such as *Escherichia coli*, *Pseudomonas aeruginosa*, *Staphylococcus aureus*, and *Bacillus* spp. They also showed the highest antifungal effect against *A. flavus* and the lowest effect against *Penicillium griseofulvum*. Das et al. [84]. reported that the surface coating of biogenic AgNPs with zinc oxide increased its antimicrobial efficiency against *Candida krusei*. AgNPs produced by propolis extract-mediated synthesis exhibited antifungal activities against various fungi, including *Candida* sp., *Fusarium* sp., *Microsporum* sp., and *Trichophyton* sp. AgNPs also acted synergistically with fluconazole to inhibit the growth of fluconazole-resistant *C. albicans* by abrogating drug efflux pumps and increasing endogenous ROS production. Treatment with AgNPs downregulated ERG1, ERG11, ERG25, and CDR2, decreased membrane ergosterol levels and membrane fluidity. It also reduced the membrane content of Cdr1p and Cdr2p, and thus efflux pump activity. A combination of Ag-, Au-, metallic- and ZnO-NPs effectively controlled the growth of four mycotoxin-producing mold strains, including *A. flavus* and *A. fumigatus* [85]. Treatment with AgNPs significantly inhibited the growth of six fungal species, including *C. albicans*, *C. glabrata, Candida parapsilosis, C. krusei,* and *Trichophyton mentagrophytes* by altering the structure of the cell membrane and inhibiting the normal budding process [86,87,88]. Monodispersed monometallic and bimetallic nanoparticles, which are highly reactive NPs with sizes less than 5 nm, exhibited high antifungal activity against *C. parapsilosis*, *C. krusei*, *C. glabrata*, *Candida guilliermondii*, and *C. albicans* [89]. Immobilized AgNPs on chitosan also enhanced antifungal efficacy against *Monilia albicans* [90]. In addition, a nanocomposite containing pullulan and AgNPs exhibited antifungal activity against *Aspergillus niger* [91] Several studies demonstrated that AgNPs can act as potential antifungal agents, although this depends on their sizes, shapes, and zeta potentials. Moreover, the mechanism of inhibition of fungi varies with particle size. AgNPs were found to be effective against various fungal pathogens, including *C. albicans*, *Candida tropicalis*, *Trichophyton rubrum*, *Penicillium brevicompactum*, *Cladosporium cladosporioides*, *A. fumigatus*, *Chaetomium globosum*, *Mortierella alpina*, and *Stachybotrys chartarum* [19,92,93,94]. A different study was conducted to determine the efficacy of AgNPs against various fungal pathogens that cause fungal keratitis, a vision-threatening infection. AgNPs exhibited antifungal activity with minimal inhibitory concentrations of 1, 0.5, and 0.5 μg/mL for *Fusarium* spp., *Aspergillus* spp., and *Alternaria alternata*, respectively [95]. Increasing the concentration of Ag-embedded mesoporous silica nanoparticles (mSiO2@AgNPs) using a leaf extract of *Azadirachta indica* improved its potential antifungal activity against *C. albicans* [96]. AgNPs functionalized with phenolic compounds also showed potential antifungal effects against *A. niger* by interfering with spore germination and mycelial growth. Biologically synthesized ultrasmall silver nanoclusters (rsAg@NCs) using metabolites from usnioid lichens exhibited excellent antimicrobial activity against fluconazole (FCZ)-resistant *C. albicans*, which was significantly higher than that of chemically synthesized silver nanoparticles (AgNPs) and FCZ. The rsAg@NCs induced apoptosis via ROS generation, which leads to the loss of mitochondrial membrane potential, DNA fragmentation, chromosomal condensation, and metacaspase activation [97]. AgNPs synthesized by *Mucor hiemalis* showed significant antibacterial activity against six pathological bacterial strains (*Klebsiella pneumoniae, Pseudomonas brassicacearum, Aeromonas hydrophila, E. coli, Bacillus cereus*, and *S. aureus*) and three pathological fungal strains (*C. albicans, Fusarium oxysporum*, and *A. flavus*). AgNPs were found to synergistically inhibit bacterial growth when used in combination with several antibiotics (streptomycin, tetracycline, kanamycin, and rifampicin). In addition, they were found to inhibit the growth of fungi when used in conjunction with several fungicides (amphotericin B, fluconazole, and ketoconazole). AgNPs synthesized using aqueous *Eucalyptus camaldulensis* showed strong fungicidal activity ranging from 0.5 to 1 µg/mL affecting *Candida* adhesion and invasion into host cells by reduced germ tube formation and hydrolytic enzyme secretion. Furthermore, AgNPs downregulated the expression of *ALS3*, *HWP1*, *ECE1*, *EFG1*, *TEC1*, and *ZAP1*, which encode proteins required for hyphal growth and biofilm development, and PLB2, LIP9, and SAP4, which encode hydrolytic enzymes [98]. AgNPs synthesized using *Cinnamomum camphora* fruit extract exhibited excellent antifungal effects against *F. oxysporum* by inhibiting colony growth and conidia germination [13]. The metal nanoparticles (MNPs) synthesized by *Fusarium solani* showed also showed potential antifungal effects against multidrug-resistant (MDR) *P. aeruginosa* and *S. aureus* in addition to its mycotoxigenic activity against *Aspergillus awamori*, *A. fumigatus,* and *F. oxysporum* [99]. 

## 3. Zinc Oxide Nanoparticles

Fungal pathogens present a major health problem and can cause severe damage. Chitosan (CS) functionalized with linoleic acid (LiA) quantum dots inhibited the growth of fluconazole-resistant clinical strains at concentrations similar to those of fluconazole [100]. ZnO NPs inhibit the viability of *C. albicans* in a concentration-dependent manner. The minimal concentration of ZnO NPs (0.1 mg/mL) inhibited the growth of *C*. *albicans* by over 95%. The loss of viability was reduced in the presence of histidine, suggesting the involvement of reactive oxygen species, including hydroxyl radicals and singlet oxygen, in cell death [101]. Nano-ZnO doped with 5% nano-Pd, pure nano-ZnO, and micro-ZnO, showed antifungal activity against *A. niger* with minimum inhibitory concentrations (MICs) of 1.25, 2.5, and 5 mg/mL, respectively. Among the three tested compounds, nano-ZnO exhibited a significantly stronger induction of cell death in *Aspergillus* compared to its non-nano-counterpart [102]. ZnO QDs at concentrations higher than 3 mmol/L inhibited the growth of *P. expansum* [103]. ZnO NPs embedded within a mesoporous nanosilica (mSiO_2_) matrix (ZnO@mSiO_2_) inhibited the growth of four strains of fungi in a dose-dependent manner. ZnO NPs remarkably reduced germ tube formation in *C. albicans* by decreasing phospholipase and proteinase secretion. ZnO NPs penetrate the cell and cause extensive damage to the cell wall and cell membrane [104]. Fungal keratitis (FK) is caused by pathogenic bacterial and fungal strains that contaminate contact lenses. Zinc oxide nanoparticles functionalized with gallic acid (GA) exhibited antifungal activity against various types of fungal strains, including *C. albicans*, *A. fumigatus*, and *F. solani* [105]. The growth inhibition of *Penicillium oxalicum*, *Pestalotiopsis maculans*, *Paraconiothyrium* sp., and *A. niger* fungi achieved by Mg1x ZnxO QDs was higher than that obtained by either of the two pure oxide QDs [106]. ZnO QDs time- and dose-dependently inhibited radial growth against two different types of fungi, such as *A. fumigatus* and *C. albicans* [107]. Cierech et al. [108]. reported the antifungal properties of PMMA and doped ZnO NPs were mediated by the inhibition of biofilm deposition on the surface in a concentration-dependent manner. *Syzygium aromaticum* functionalized ZnO NPs (SaZnO NPs) reduced the growth and production of deoxynivalenol and zearalenone in *Fusarium graminearum* through the accumulation of ROS in a dose-dependent manner. Furthermore, SaZnO NPs treatment enhanced lipid peroxidation, depleted ergosterol content, and caused detrimental effects on fungal membrane integrity [109].

## 4. Gold Nanoparticles

Gold nanoparticles (AuNPs) have unique properties and are not inherently toxic to human cells, which makes them promising therapeutic agents [110,111,112]. AuNPs have been widely used in cancer treatments as a drug delivery system and in thermal therapy [113,114]. In addition, AuNPs have shown potential activity against various pathogens, including *E. coli*, *Streptococcus mutans*, and *Candida* species [34,115,116]. Khan et al. [117]. reported that antibiofilm assays and microscopic studies showed that gold nanoparticle-methylene blue conjugates significantly reduced biofilm formation [117]. The shape of nanoparticles plays a significant role in their antimicrobial activity. To demonstrate the impact of the shape of the silver and gold nanoparticles, nanoparticles of different sizes were selected, including nanocubes, nanospheres, and nanowires, and their effect on *C. albicans*, *C. glabrata*, and *C. tropicalis* was assessed. The study found that silver and gold nanocubes had higher antifungal properties against the test species than did nanospheres or nanowires [118]. AuNPs directly interacted with DNA and led to nuclear condensation and DNA fragmentation. AuNPs can cause an overload of mitochondrial Ca^2+^ and the collapse of mitochondrial Ca^2+^ homeostasis. Thus, the disruption of mitochondrial Ca^2+^ homeostasis could play a role in initiating AuNP-induced cell death in *C. albicans* [119]. AuNPs have been shown to strongly inhibit pathogenic biofilm formation and the invasion of dental pulp stem cells (DPSCs). Moreover, treatment with AuNPs led to the activation of immune response-related genes in DPSCs, which could enhance the activity of the host immune system against pathogens [120]. Antimicrobial activity was evaluated using stabilized cationic dipeptide-capped gold/silver nanohybrids, in which peptide-capped AgNPs exhibited higher antimicrobial activity than peptide-capped AuNPs, native peptides, or unconjugated metallic nanoparticles [121]. AgNPs and AuNPs were synthesized using sodium borohydride and tannic acid as reducing agents, and then both were tested for antifungal activity against *C*. *albicans* and *Saccharomyces cerevisiae*. Of the two reagents, AgNPs showed greater potential antifungal activity compared to that of AuNPs [122]. Quercetin-functionalized AuNPs showed significant antioxidant activity compared to quercetin alone. It also exhibited significant antifungal activity against *A. fumigatus* at concentrations ranging from 0.1 to 0.5 mg/mL [123]. Heparin-functionalized gold nanoparticles (AuHep-NPs) with an average size of 530 nm also showed significant antifungal activity against *C. albicans*, *Issatchenkia orientalis* (*C. krusei*), and *C. parapsilosis* [124]. AuNPs coated with the antimicrobial peptide indolicidin inhibited biofilm formation and development in clinical isolates of *Candida* spp. [46]. A further study suggested that indolicidin-conjugated AuNPs exhibited potential antifungal effects against fluconazole-resistant strains of *C. albicans* isolated from patients with infected burns [125].

## 5. Copper Oxide Nanoparticles

Copper has been widely used as an antimicrobial agent. Several studies have demonstrated the antimicrobial activity of copper nanoparticles (CuNPs) against multiple fungi [126,127,128]. Oussou-Azo et al. [129]. reported that all the forms of copper they studied inhibited hyphal growth in a dose-dependent manner. They also reported that CuNPs and CuO NPs showed the greatest inhibitory effects against fungal pathogens. Cu NPs are considered effective in inhibiting the growth and colony formation of *Colletotrichum gloeosporioides*. The toxicity of copper particles depends on a combination of several factors, such as concentration, length of exposure, humidity, and temperature. Copper induces cell death by interacting with microorganisms, and it can alter cell membrane permeabilization, membrane lipid peroxidation, protein alteration, and denaturation of nucleic acids, which ultimately leads to cell death. Surface coatings of copper are also able to provide antifungal effects [30,130,131,132]. The CuO NPs exhibited maximum antibacterial and antifungal activities against human clinical pathogens by the generation of free radicals, especially nitric oxide radicals [133]. In addition, decorating Ag NPs with binary silver/copper nanoparticles increased the antibacterial and antimycotic activity of cotton fabrics. Textile materials containing ANPs demonstrated high antibacterial activity, whereas fabrics doped with bimetallic composite Ag/Cu had pronounced antimycotic properties [134]. CuO NPs with a spherical shape and an average size between 20 and 50 nm were shown to exert antifungal activity against *Fusarium* sp. at a concentration of 450 ppm. After a nine-day incubation at this concentration, 93.98% of fungal growth was inhibited [135]. Green synthesis of CuNPs resulted in CuNPs that had antifungal effects against *F. solani*, *Neofusicoccum* sp., and *F. oxysporum*. These CuNPs acted by damaging the cell membranes of the pathogens and by inducing the intracellular generation of ROS in the mycelium [136]. CuO NPs synthesized using *Cissus quadrangularis* plant extract had an average size of 30 ± 2 nm. The synthesized CuO NPs exhibited dose-dependent potential activity against *A. niger* [137]. Copper oxide nanocomposites (CuO/C nanocomposites) with a particle size of 50 nm have also been prepared using sucrose as a capping agent. CuO/C nanocomposites exhibited potential antifungal activity against *A. niger* and *A. flavus* species [138]. CuNPs with a size range of 23–82 nm and a round to polygonal shape also exhibited potential antifungal activity against rotting plant pathogens, such as *F. oxysporum and Phytophthora parasitica* [139]. CuO NPs synthesized using green and black tea leaf extracts containing flavonoids and phenols that have a spherical shape and an average size between 26 and 40 nm also exhibited antibacterial and antifungal activities. Synthesizing CuO NPs using *Persea americana*-derived seed extracts that contained carboxylic acid alkanes resulted in CuO NPs with a spherical shape and an average size from 42 to 90 nm. These CuO NPs also showed antibacterial, antifungal, and antioxidant activities [140]. Microwave-mediated synthesis of CuO NPs resulted in CuO NPs that were spherical in shape and had an average diameter of 7–10 nm and that showed significant antifungal activity against *Cladosporium herbarum* [141]. Aqueous-phase mediated synthesis of nanoparticles, such as copper/copper oxides, also resulted in NPs that showed antifungal effects against *F. oxysporum*. Both of these nanoparticles showed a high percentage of inhibition of radial growth (IGR) [142]. CuO NPs significantly induced cell death in *C. albicans* by stimulating the production of ROS and by causing membrane damage. This damage eventually leads to the suppression of yeast-to-hyphae morphological switching by downregulating *cph1*, *hst7,* and *ras1* and by upregulating the negative regulator *tup1* [143]. Martínez et al. [144]. evaluated the in vitro activity of CuNPs and copper nanowires against *C. albicans* strains and yeast. Microscopic analysis revealed that treatment with CuNPs and nanowires altered cell morphology and cell wall, which ultimately leads to the complete collapse of the yeast. When *C. albicans* was exposed to CuNPs and nanowires, the nanoparticles and nanowires exhibited potential antifungal activity by releasing free Cu^2+^ ions that acted as a biocide. In addition, the sharp edges of marigold-like petal nanostructures were able to injure the cell wall and membrane and cause the death of the yeast. The starch-and sodium alginate-mediated synthesis of CuNPs resulted in nanoparticles that showed antifungal activities against *C. albicans* and *C. krusei* [145]. Silver and CuO NPs significantly reduced the mycelial growth of *A. alternata* and *P. oryzae* in a dose-dependent manner [146].

## 6. Titanium Dioxide Nanoparticles

Titanium dioxide nanoparticles (TiO_2_ NPs) have a wide range of applications in industry and medicine, including in drug delivery systems and water purification. TiO_2_ NPs have unique optical, electronic, photocatalytic, and antimicrobial properties [147]. *Candida* species are known to cause serious cutaneous, mucosal, and systemic fungal infections. TiO_2_ NPs in their anatase and rutile forms displayed significant antifungal activity against *C. albicans*. The loss of cell viability was dose- and time-dependent. TiO_2_ NPs also alter cell morphology [148]. Due to their high photocatalytic activity, photocatalytic paints formulated with the anatase form of TiO_2_ exhibited antifungal effects against *A. niger*. Several studies have reported the antifungal activity of photocatalytic paints using *A. niger* as an environmental model, along with non-pathogenic molds [149,150]. Moradpoor et al. [151]. reported that TiO_2_ NPs exhibited significant antifungal activities against oral *C. albicans* pathogens. N and F co-doped TiO_2_ nanoparticles with sizes of 200–300 nm were used as antifungal agents under visible light. The colloidal form, with its appropriate surface chemistry, allows the efficient interaction of the nanoparticles with the fungal cell wall, and visible light-induced ROS generation by the nanoparticles results in fungal disinfection [152].

A study was conducted to compare the antifungal activities of TiO_2_ NPs and ZnO NPs with those of amphotericin B (AmB) against different species of *Candida*. The results showed that TiO_2_ and ZnO NPs exhibited antifungal activity against pathogenic *Candida* species. The results revealed that TiO_2_ and ZnO NPs were significantly less active than AmB [153]. In the same study, antifungal activity was evaluated using various types of nanoparticles, such as ZnO, TiO_2_, and ZnO–TiO_2_, by the sol-gel method. All synthesized nanoparticles (50 μg/mL) showed fungal growth inhibition, and ZnO–TiO_2_ showed higher antifungal activity against *A. flavus* than pure TiO_2_ or ZnO. In combination, ZnO and TiO_2_ showed potential antifungal activity at a low concentration (150 μg/mL) of ZnO–TiO_2_ and exhibited higher ROS production and induction of oxidative stress [154]. UV-irradiated TiO_2_ NPs attack microbial phospholipids, thereby causing cell damage [155]. TiO_2_ released ROS upon direct contact with the cell membranes of bacterial cells. TiO_2_ NPs have a positive charge on their surface, and microbial cell membranes have a negative charge, which enhances their attachment [156]. TiO_2_ NPs show a high affinity for membrane proteins and can alter their structures. Photocatalysis also influences the ability of TiO_2_ NPs to bind with phosphoproteins and phospholipids [157,158]. Under UV light, the electron holes in nano-TiO_2_ interact with water and oxygen to generate active oxygen species, especially hydroxyl radicals, thereby achieving antimicrobial effects [159]. TiO_2_-coated film significantly inhibited the development of *Penicillium* rot in apples through a photocatalytic reaction and reduced the risk of microbial growth on fresh-cut produce [160,161]. Chitosan nanoparticles detrimentally affect the function of the cell membrane and exhibit antibacterial properties. Increasing the concentration of chitosan leads to the degradation of chitin, thus destroying the cell wall of the pathogen and achieving an antifungal effect [162,163]. Chitosan and TiO_2_ composites can adsorb onto the cell surface well and easily penetrate the cell wall, thereby causing the leakage of intracellular material and impaired nuclear function. this results in the inhibition of cell growth or death. Similarly, the oxygen free radicals produced can attack the outer membrane, DNA, RNA, and lipids. This oxidation can damage bacteria, leading to the inhibition of cell growth or death [164,165,166]. Chitosan can alter the permeability of a fungal cell membrane, resulting in extensive electrolyte leakage and, consequently, cell death [167]. Nano-TiO_2_ treatment produces strong oxides, such as hydroxyl radicals, which can destroy the unsaturated bonds in organic molecules, damage cell components, break the cell wall membrane, and destroy the permeable barrier, all of which ultimately result in cell death [168,169]. 

## 7. Magnetic and Iron Oxide Nanoparticles

Magnetic nanoparticles of iron oxides (Fe_3_O_4_ and/or γ-Fe_2_O_3_) are currently used as contrast agents in magnetic resonance imaging (MRI) studies and cancer treatments [170]. Nehra et al. [171]. investigated the antibacterial and antifungal activities of bare and chitosan-coated Fe_3_O_4_ NPs against five organisms. The results showed that chitosan-coated Fe_3_O_4_ NPs could be potentially effective antimicrobial agents against various microorganisms, such as *E. coli, Bacillus subtilis, C. albicans, A. niger, and F. solani.* Biologically synthesized Fe_2_O_3_-CsNPs significantly inhibit the Verticillium wilt pathogen *Verticillium dahlia* by altering the morphology of its hyphae [172]. A study was performed to evaluate the antifungal activity of IONPs against different species of *Candida*. compared with fluconazole (FLC). The results suggested that IONPs can potentially inhibit the growth of *C. tropicalis*, *C. albicans,* and *C. glabrata* [173]. One study reported that incubation of fungal cells with MNPs inactivates the catalase Cta1 and disturbs the oxidation-reduction balance [174]. Furthermore, studies suggest that MNPs coated with LL-37 or CSA-13 were able to increase the generation of ROS, which is probably associated with the mechanism of action of CAPs, which results in pore formation within cell membranes. The formation of these pores, contributes to nanoparticle transport into *Candida* cells. Excessive ROS production can destroy the integrity of the fungal cell wall, which enhances MNP internalization and oxidative damage [175]. Unmodified nanoparticles strongly inhibit *C. albicans* biofilm formation due to cell wall disruption, resulting in the loss of fungal biofilm structure [176]. Magnetite nanoparticle-decorated reduced graphene oxide showed antifungal activities against *T. mentagrophytes* and *C. albicans*. Hence, rGO/Fe_3_O_4_ NCs are used as excellent adsorbents to remove organic dye pollutants and kill pathogens. 

IONPs are of immense interest due to their superparamagnetic properties and their potential biomedical applications, which arise from their biocompatibility and non-toxicity [177]. IONPs have various applications, including drug and gene carriers for target-specific drug delivery and gene therapy, respectively. The surface coating of IONPs not only decreased their cytotoxicity, but also increased the stability and efficiency of the antimicrobial potential of IONPs [178]. IONPs synthesized using *P. orientalis* showed significant antifungal activities against *Mucor piriformis* as compared with *A. niger* [179]. Salari et al., (2018) evaluated the biofilm formation ability of different *Candida* strains and the antibiofilm effects of Fe_3_O_4_-NPs compared with fluconazole (FLC). After exposure to various concentrations of Fe_3_O_4_-NPs, the ability of the nanoparticles to reduce biofilm formation in *C. albicans* and *C. parapsilosis* was greater than that of FLC. The iron oxide nanoparticles (Fe_2_O_3-_NPs) in the study had sizes of 10–30 nm and were prepared using a green approach that used tannic acid as a reducing and capping agent. The effect of the prepared nanoparticles was evaluated using *Trichothecium* *roseum,* *C. herbarum,* *Penicillium chrysogenum,* *A. alternata,* and *A. niger*. Among the tested fungi, Fe_2_O_3-_NPs were found to be the most effective against *P. chrysogenum* and *A. niger*. Fluconazole (FLZ) on chitosan (CS)-coated IONPs potentially inhibits *Candida* planktonic cells [180]. 

## 8. Carbon Nanoparticles

**Figure 1:**
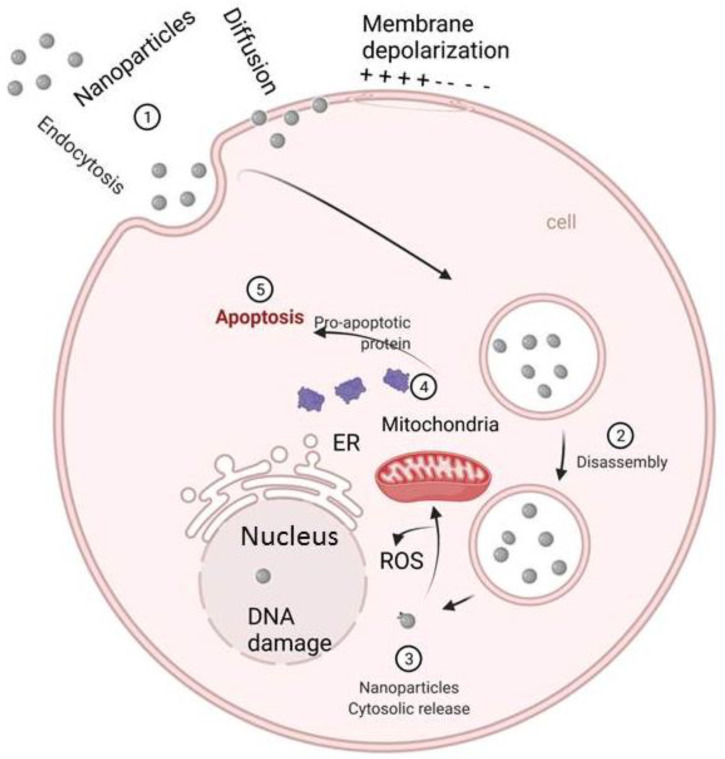
Mechanism of antimicrobial activity of nanoparticles against fungi Nanoparticles inhibit fungal growth by entering the cells through endocytosis and diffusion processes, and their entry will eventually lead to apoptosis. After entering into the cells, nanoparticle inhibits cell wall/membrane synthesis, disruption of energy transduction, production of ROS, enzyme inhibition, and reduced DNA production, DNA damage, and protein damage.

Graphene oxide (GO) is a new nanomaterial with different potential biomedical applications due to its excellent physicochemical properties and ease of surface functionalization. Carbon nanoscrolls (CNSs) filled with AgNPs exhibited ideal lengthened activities against *C. albicans* and *C. tropicalis* when compared with the GO-AgNPs nanocomposites [181]. Cationic fullerene derivatives bearing a substituted-quinazolin-4(3H)-one moiety exhibited significant activity against various fungal strains, including *C. albicans, A. clavatus*, and *A. niger* [182]. Polyethelene glycol functionalized GO potentially inhibits cell viability and can control fungal infections by limiting the growth of the opportunistic fungal pathogen *C. albicans* and activating immune effector cells. Thus, it can prevent invasive candidiasis [183]. Size-controllable red-emissive carbon dots (CDs) with positively charged guanidine groups (Gu^+^) and conjugated with the antifungal polyene AmB exhibited potent antifungal effects in both the planktonic and biofilm forms [184]. The antibacterial and antifungal activity of CDs derived from tamarind arises from its interaction with DNA via intercalation [185]. Their antifungal activity against *C. albicans* was evaluated using a novel hydrophilic nanoconjugate gold@carbon dot (Au@CD), among others. The nanoconjugates exhibited a profound effect on the susceptibility and size of *C. albicans* cells [186]. Reduced graphene oxide (rGO) inhibited the mycelial growth of several fungal pathogens, including *A. niger, Aspergillus oryzae, and F. oxysporum* [187]. Single-walled carbon nanotubes (SWCNTs), multi-walled carbon nanotubes (MWCNTs), fullerene (C_60_), and rGO all show significant antifungal activity against *F. graminearum* and *Fusarium poae* [188]. Graphene sheets also showed potential antifungal effects by disturbing fungal structures and damaging the cell wall with their sharp edges (Xie et al., 2016) [189], producing oxidative stress [190]. Nanoparticles inhibit fungal growth by entering the cells through endocytosis and diffusion processes, and their entry will eventually lead to apoptosis. The mechanism of antifungal activity of various types of NPs includes five different steps: (1) internalization of NPs through endocytosis and diffusion process, (2) disassembly of the membrane, (3) release of nanoparticles into the cytoplasm, (4) inducing oxidative stress, and (5) ROS induced cell death by the activation of pro-apoptotic proteins and ROS production, which leads to mitochondrial dysfunction and the endoplasmic reticulum stress-mediated apoptosis pathway (Figure 1). Miragoli et al., (2013) reported that positively charged, amine-modified polystyrene latex nanoparticles induced large-scale damage to cardiomyocyte membranes leading to calcium alternans and cell death. Conversely, negatively charged, carboxyl-modified polystyrene latex nanoparticles (NegNPs) triggered the formation of 50–250-nm nanopores in the membrane. In vitro study demonstrated that when ventricular myocytes were exposed to TiO_2_ they had significantly reduced action potential duration, impairment of sarcomere shortening, and decreased stability of resting membrane potential [191]. Furthermore, in vivo studies depicted that TiO_2_ nanoparticles increased cardiac conduction velocity and tissue excitability, resulting in an enhanced propensity for inducible arrhythmias [192]. An antifungal effect of representative nanoparticles is summarized in Table 1 [136,193,194,195,196,197,198,199,200,201,202,203,204,205,206,207,208,209,210,211,212,213,214]. 

**Figure 2:**
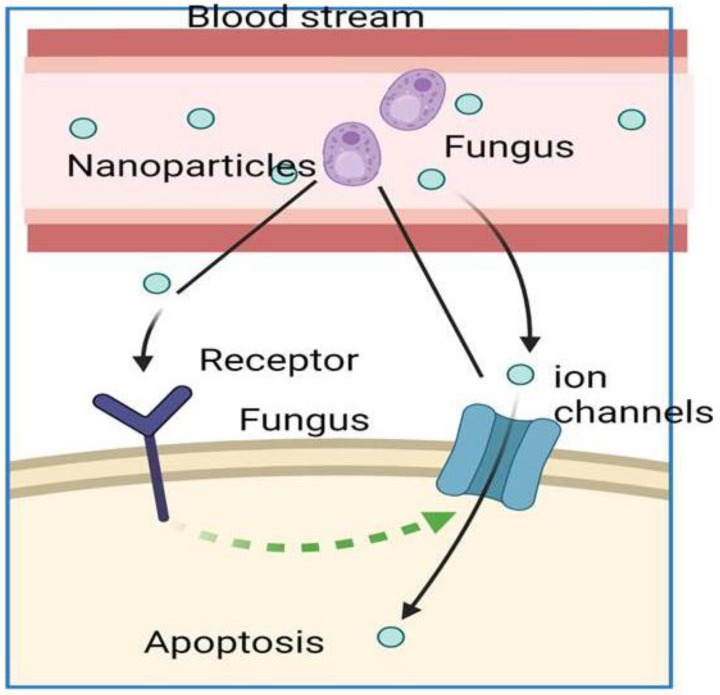
Fungi penetrating the blood-brain barrier and circulating in the bloodstream. Fungal propagation involves spore inoculation, escaping phagocytosis by macrophages and neutrophils, hyphae germination, growth by taking advantage of the condition of the host attaching to the endothelium using receptors, and damaging the endothelium and eventually causes impairs ion channels and induces apoptosis. This process results in tissue necrosis, thrombus formation, or hemorrhage.

Mucormycosis is caused by various saprophytic fungi in the order of Mucorales, including members of the *Rhizopus*, *Lichtheimia*, *Mucor*, and *Rhizomucor* genera [215]. These fungi affect various organs, including the lungs, skin, gut, rhinocerebral areas, and central nervous system [216]. *Rhizopus arrhizus* is the most common causative agent globally, and *Apophysomyces* is the second most common species to cause mucormycosis [217]. Generally, these fungi are relatively larger than other species and thus are easily retained in the paranasal sinuses. However, certain smaller species may also reach the lungs [218]. Usually, the spores of these fungi disperse through the air and enter humans via inhalation, inoculation in skin lesions and other similar areas, and orally, which allows them to enter the gastrointestinal tract [219]. Fungal propagation involves spore inoculation, escaping phagocytosis by macrophages and neutrophils, hyphae germination, growth by taking advantage of the condition of the host (such as an iron overload or ketoacidosis), attaching to the endothelium using receptors, and damaging the endothelium. This process results in tissue necrosis, thrombus formation, or hemorrhage (Figure 2). All of these conditions contribute to multiorgan dysfunction. The spores enter into the human body by inhalation, ingestion, or direct inoculation through wounds, and then germinate angioinvasive hyphae [220]. Fungi enter the human body through adherence to the extracellular matrix proteins present in the basement membrane, laminin, and collagen VI by specific binding and subsequently secrete lipolytic/glycosidic enzymes and proteases that degrade the underlying stroma, ultimately facilitating fungal invasion into the host tissues [221,222]. 

The pathogenesis of mucormycosis includes the process of thrombosis, in which the fungi adhere to the endothelial cells and disrupt their integrity so as to gain access to the bloodstream [223]. Fungal spores bind to coat homolog proteins on their surface which enables the spores to effectively bind by means of adhesins to 78-kDa glucose regulator protein 78 (GRP78) receptors present in the endothelial cells [222]. Upregulation of GRP78, a heat shock protein due to increased glucose and iron content caused by diabetic ketoacidosis (DKA) or induced by the use of dexamethasone, also facilitates fungal entry [224]. This is especially true for patients with COVID-19. After binding to the blood vessels, secondary metabolites released during the phagocytosis of *R. oryzae* by the endothelial cells are the cause of the disruption to the endothelial lining, and not viable fungi [225]. Hyperglycemia is a significant risk factor for mucormycosis. COVID-19 infection may directly induce hyperglycemia by damaging pancreatic beta cells or by the use of corticosteroid therapy, which may contribute to CAM pathogenesis. GRP78 receptors not only play a role in the pathogenesis of COVID-19 but are also involved in mucormycosis pathogenesis. Increased expression of the GRP78 receptor may also enhance the binding of Mucorales spores, resulting in enhanced endothelial invasion and damage in patients with COVID-19 [226,227].

Mucormycosis significantly affects patients with COVID-19 in three ways. COVID-19 leads to a reduction in the availability of T cells, an increase in pro-inflammatory markers in patients with severe disease, and pronounced damage to the pulmonary tissues. All of these changes aid in fungi invasion [228,229]. The cytokine storm caused by COVID-19 increases the level of IL-6, which can lead to high levels of iron and macrophage activation. This can result in the increased availability of free iron within cells. These free iron species support the growth of fungi. This, in turn, causes endothelial destruction and inflammation, which is called endothelitis [224]. Thus, COVID-19–induced immunosuppression increases the risk of opportunistic infection, damages the endothelium and alveolae (which makes fungal invasion easier), and increases the glucose level due to the acute diabetes-like state caused by pancreatic damage. The increased ferritin level also supports fungal growth, and an increase in body temperature is optimal for thermotolerant fungi. In patients with uncontrolled diabetes and poor glycemic control, ketone concentrations in the blood increase, which leads to acidosis [227,230]. 

Iron plays a significant role in mucormycosis infection in patients with COVID-19. Iron-binding proteins, such as transferrin, ferritin, and lactoferrin, limit the release of free iron in the blood and the maintenance of iron homeostasis [231]. Patients with diabetes mellitus show increased serum ferritin levels, which leads to increased iron stores [232]. In patients with COVID-19, SARS-CoV-2 interacts with the hemoglobin molecule and causes the dissociation of iron from heme molecules, which leads to hyperferritinemia in patients with COVID-19 [233]. Increased levels of serum ferritin, IL-6, and D-dimers have been associated with high mortality in patients with COVID-19 [234]. Hyperferritinemia in patients with COVID-19 leads to increased lung inflammation and lung fibrosis, leading to severe disease [235]. Free iron in the blood facilitates the growth and invasion of blood vessels, causing vessel thrombosis and tissue necrosis [219]. 

## 9. Impact of Nanoparticles on Black Fungus

Coronaviruses are a diverse group of viruses that infect many different animals and can cause mild-to-severe respiratory infections in humans. Among the various types of coronaviruses, SARS-CoV-2 and Middle East respiratory syndrome coronavirus (MERS-CoV) caused pathogenic diseases in 2002 and 2012, respectively [236]. COVID-19 is caused by severe acute respiratory syndrome coronavirus 2 (SARS-CoV-2) and is an ongoing global health emergency [237,238]. SARS-CoV-2 causes an ongoing outbreak of the disease in the lower respiratory tract. SARS-CoV-2 is a highly transmissible and pathogenic coronavirus that emerged in late 2019 and has caused a pandemic of acute respiratory disease, named ‘coronavirus disease 2019′ (COVID-19), which threatens human health and public safety. As of 15 June 2021, there have been 175,987,176 confirmed cases of COVID-19, and 3,811,561 deaths have been reported worldwide. It has affected at least 210 countries. Chan et al. [239]. reported evidence of human-to-human transmission of SARS-CoV-2. SARS-CoV-2 shares 79% genome sequence identity with SARS-CoV and 50% with MERS-CoV [237]. SARS-CoV-2 has six functional open reading frames (ORFs), which are listed in order from 5′ to 3′: replicase (ORF1a/ORF1b), spike (S), envelope (E), membrane (M), and nucleocapsid (N). SARS-CoV-2 uses angiotensin-converting enzyme 2 (ACE2) for infection [240]. SARS-CoV-2 infects cells through proteolytic processing of the S protein, which activates the entry of SARS-CoV-2, and involves transmembrane protease serine protease 2 (TMPRSS2), cathepsin L, and furin [241,242,243]. The pathogenesis of SARS-CoV-2 begins when it binds to epithelial cells in the respiratory tract. After that, SARS-CoV-2 starts replicating and migrates down into the airways, where it enters alveolar epithelial cells in the lungs, initiates replication, and eventually triggers a strong immune response. The replication of SARS-CoV-2 can induce cytokine storm syndrome, which causes acute respiratory distress syndrome and respiratory failure, ultimately resulting in death [13,244,245,246,247,248]. Recently, it has been found that COVID-19 infection results in a weakened immune system, which makes it harder for the body to effectively prevent infection. As a result, COVID-19 patients are at risk for mucormycosis, which is a rare form of infection. 

Mucormycosis is an uncommon invasive fungal infection. It is a destructive, life-threatening infection that occurs in people with a susceptible immune system, including people with uncontrolled diabetes mellitus, people who have low levels of neutrophils, or those who are undergoing immunosuppression as part of their treatment for blood cancer, hematopoietic stem cell transplantation, or solid-organ transplantation [249]. The most common symptom is a sinus infection (sinusitis) that is accompanied by nasal congestion, nasal discharge, and sinus pain. Sometimes, blurry vision or double vision can develop, followed by vision loss. Examples of mucormycosis-causing fungi include *Rhizopus* spp., *Mucor* spp., *Rhizomucor* spp., *Syncephalastrum* spp., *Cunninghamella bertholletiae*, *Apophysomyces* spp., and *Lichtheimia* spp. One study that involved *Zataria multiflora* essential oil (ZEO) and *Zataria multiflora* essential oil-loaded solid lipid nanoparticles (ZE-SLNs) with an average size of 255.5 ± 3 nm showed that the ZEO and ZE-SLNs inhibited the growth of *Aspergillus ochraceus*, *A. niger*, *A. flavus*, *Alternaria solani*, *Rhizoctonia solani*, and *Rhizopus stolonifera* by 54 and 79%, respectively [250]. Schiff base ligand 2-(4,6-dimethoxypyrimidin-2-ylimino)methyl)-6-methoxyphenol (DPMM) stabilized copper nanoparticles (DPMM-CuNPs) and nickel nanoparticles exhibited dose-dependent antibacterial and antifungal effects. Studies of the activity of DPMM-CuNPs and DPMM-NiNPs against *Shigella sonnei*, *S. aureus*, *E. coli*, *K. pneumoniae*, *Pseudomonas fluoroscens*, and *A. niger*, *C. albicans*, *C. tropicalis*, *Mucor indicus* and *Rhizopus* spp. [251]. ZnONPs significantly inhibited the growth of three postharvest fungal isolates including *A. alternata*, *Rhizopus stolonifer*, and *Botrytis cinerea* in a dose-dependent manner [252] *Saussurea lappa* plant root mediated ZnO NPs with an average size of 123.5 nm also showed significant antifungal activity against *A. niger*, *A. flavus, F. oxysporum,* and *Rhizopus oryzae* [253]. The spherical shape of AgNPs synthesized using the aqueous extract of red seaweed (*Gelidiella acerosa*) showed antifungal effects against *Humicola insolens* (MTCC 4520), *Fusarium dimerum* (MTCC 6583), *Mucor indicus* (MTCC 3318) and *Trichoderma reesei* (MTCC 3929). *Mucor hiemalis*-derived AgNPs exhibited activity against six pathological bacterial strains, namely *K. pneumoniae, P. brassicacearum, A. hydrophila, E. coli, B. cereus*, and *S. aureus,* and three pathological fungal strains, namely *C. albicans, F. oxysporum*, and *A. flavus*. The antimicrobial activity of AgNPs is comparable to that of antibiotics and fungicides [254]. Dilshad et al. [255]. reported that nanosized silver particles (AgNPs) and micro-sized silver particles (AgMPs) exhibited antibacterial activity against gram-positive bacteria, such as *S. aureus, Bacillus subtilis,* and *Micrococcus luteus,* and gram-negative bacteria, including *Salmonella setubal, Enterobacter aerogenes,* and *Agrobacterium tumefaciens*. Silver nanoparticles demonstrated antifungal activity by inhibiting the growth of five fungal species: *Mucorn* spp., *A. niger*, *A. flavus*, *A. fumigatus*, and *F. solani*. 

## 10. Black Fungus Co-Infection in the Context of the Global COVID-19 Outbreak

**Figure 3:**
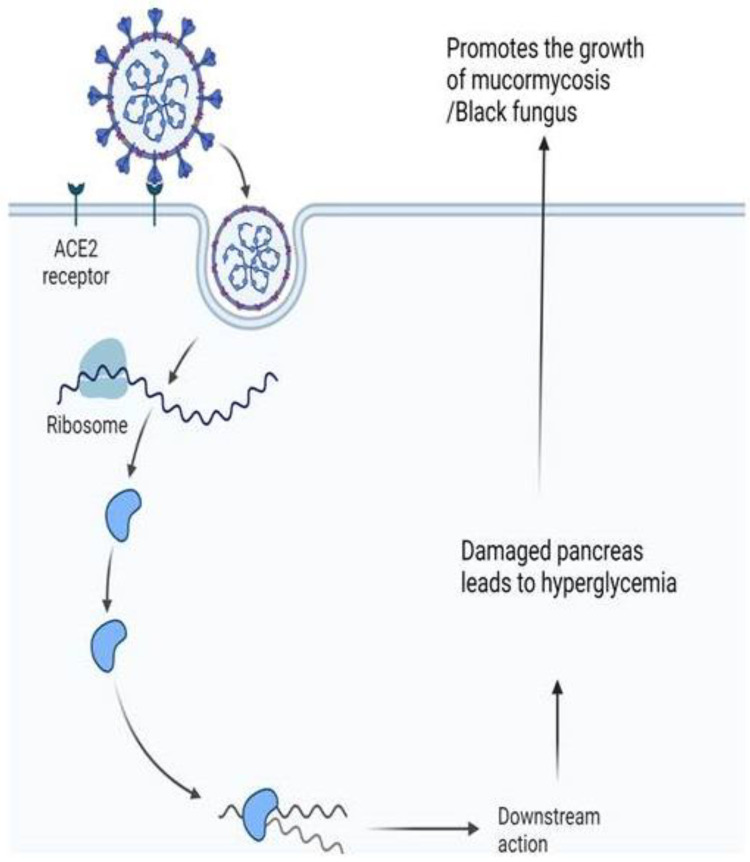
Diabetic condition promotes the growth of mucormycosis in COVID-19 patients by inducing damage to the pancreas. SARS-CoV-2 enters the human body via spike protein available on the envelope which binds with angiotensin-converting enzyme 2 (ACE 2). COVID-19-affected patients develop a dysfunction of the immune system with a decrease in lymphocyte counts and an exponential rise in inflammation. Treatment of COVID-19 patients with immunosuppressant having uncontrolled diabetes mellitus and ketoacidosis are also at major risk for Mucormycosis as it leads to dysfunctional phagocytes causing impaired intracellular killing by oxidative and non-oxidative mechanisms.

COVID-19 is linked to a variety of opportunistic bacterial and fungal diseases [256]. Aspergillosis and *Candida*-mediated infections have been identified as the primary fungal infections associated with co-infection in COVID-19 patients [9]. Recently, studies reported that mucormycosis in COVID-19 patients have been documented globally, most notably in India. Infection with COVID-19 is linked to fungal infection. Mucormycosis is an acute and deadly fungal infection caused by Mucorales-related fungal species. Uncontrolled diabetes mellitus (DM) and other immunosuppressive diseases such as neutropenia and corticosteroid treatment have been identified as risk factors for mucormycosis. Inhalation of spores that develop into angioinvasive hyphae can cause endothelial damage, resulting in local hemorrhage, thrombosis, and necrosis, as well as the eventual spread of the infection to numerous organs [220,257,258]. (Figure 3). Mucormycosis is an invasive fungal infection, often associated with extremely severe complications in immunocompromised patients [67]. COVID-19-associated mucormycosis (CAM) infections include rhinoorbital mucormycosis, pulmonary mucormycosis, and invasive fungal sinusitis. Several pathogenic fungi, such as *A. fumigatus, Rhizopus microspores, Lichtheimia ramosa,* and *R. arrhizus* have been associated with COVID-19 patients (Table 2) [259,260,261,262,263,264,265]. For instance, Iran initially found 15 CAM infections, mainly rhino-orbital mucormycosis, during the first wave of cases from April to September 2020 [67,266,267,268,269]. The rate of mortality was significantly increased due to the association between black fungus infection and COVID-19 infection [72]. The prevalence of mucormycosis (approximately 0.14 cases per 1000 population) in India is about 80 times higher than in other developed countries [217]. Overall, India reported at least 40,845 cases of mucormycosis and 3129 fatalities from such fungal agents since April 2021. Of the total number of mucormycosis patients in India, 34,940 had COVID-19, 26,187 had the co-morbidity of diabetes, and 21,523 were being treated with steroids. Substantial mortality was observed in patients with COVID-19 and diabetes ketoacidosis (DKA) [270,271,272]. Recently, a review reported 101 cases of mucormycosis in COVID-19 patients, of which 82 cases were from India. Of these patients, 18 (out of a total of 31) died due to multiple complications [272]. India has the second-highest diabetes burden in the world and has the highest death due to the disease, which may be one of the major reasons for the increasing number of deaths in patients with CAM [273]. Pakistan, Iraq, and Nepal also reported at least 22, 5, and 14 cases of CAM, respectively. 

In India, the fatality rate was 50% due to the increasing number of mucormycosis cases in many Indian states and union territories. The main cause of this issue is the overdosage, panic, and injudicious use of corticosteroids among COVID-19 patients, as well as their pre-existing medical history of diabetes. A case study suggested that most black fungus cases were in India, the United States, and Egypt. This corresponded to 73, 10, and 6% of the 99 patients, respectively. The most prevalent comorbidity was diabetes mellitus (85%). The use of glucocorticoids for the management of COVID-19 was observed in 85% of the cases. Rhino-orbital mucormycosis was most common (42%), followed by rhino-orbito-cerebral mucormycosis (24%). Pulmonary mucormycosis was observed in 10 patients (10%) [274]. Generally, black fungus infections predominantly appeared in patients whose health was compromised by the use of steroids and whose immune system was significantly weakened. An increase in cases of mucormycosis in patients with COVID-19 is prevalent, primarily due to the increased use of steroids, such as dexamethasone, especially among patients with diabetes. 

## 11. Management and Guidelines to Control COVID-19 Lead Black Fungus Infection

“Prevention is better than cure;” hence, measures are necessary to prevent COVID-19 from causing black fungal infections. First, early diagnosis via histopathology or direct microscopic examination and culture, along with molecular methods, such as sequencing common DNA region of fungi known as internal transcribed spacer (ITS) region, is essential [217]. A second important step is the proper management and treatment of patients with CAM, including the proper removal of the infected hard and soft tissue by surgery, as well as parenteral antifungal therapy with Amphotericin B.5 or antifungal drugs such as Posaconazole, Voriconazole and Itraconazole [71]. The third step is to ensure the proper and limited usage of steroid drugs for COVID-19 patients. Critical analysis is essential for the administration of drugs for fungal coinfections. The fourth step is the use of nonpharmacologic measures, such as masking policies and social distancing, which should be taken to reduce the risk of transmission of SARS-CoV-2. Vaccination is also necessary to reduce the spread of COVID-19. International efforts are needed to replenish the critical healthcare supplies and the materials required for manufacturing vaccines. The fifth step is to develop strategies to decrease the risk of mucormycosis in individuals infected with SARS-CoV-2. The sixth step is to ensure, people should avoid environments in which exposure to Mucorales is likely, and to educate healthcare providers and the public about the signs and symptoms of mucormycosis. Hospitals should also have adequate supplies of amphotericin B. It will also be necessary to make strengthening infrastructure and improving healthcare delivery systems high priorities to forestall such crises.

To restrict COVID-19 lead black fungus infections, the following criteria are essential: controlling diabetes and diabetic ketoacidosis, reducing the use of steroids, and regulating the use of immunomodulating drugs. To decrease the number of COVID-19-related black fungus infections, early screening, and testing protocols are imperative. The implementation of patient education is important because it is the best tool to prevent an increase in life-threatening mucormycotic infections. Government efforts should focus on training an ample workforce and on generating technical expertise. Epidemiological surveys must consider that COVID-19 infections can lead to black fungus infection. Other important aspects include preventing the creation of damp areas that could be a breeding ground for mucormycosis and ensuring that rapid diagnosis and treatment methods are widely available.to prevent the impact of this fungal infection. Amphotericin B is one of the best choices for the treatment of mucormycosis. Liposomal amphotericin B with a low dose of 5 mg/day to a higher dose of 10 mg/kg/day for cerebral infection [275,276]. Clinical improvement was observed with the combination of amphotericin B with triazoles such as posaconazole, isavuconazole, voriconazole, etc [277]. Caspofungin exhibited inhibitory activity against Mucorales, and a combination of amphotericin B and caspofungin showed potential synergistic effects against Mucorales. Caspofungin is effective in inhibiting the (1,3)-β-D-glucan synthetase enzyme expressed by *R. oryzae* [278]. In addition to chemotherapy, adjunctive therapies can be promoted to control mucormycosis infection, such as the administration of iron chelators and the use of hyperbaric oxygen to suppress the growth of mucormycosis-causing fungi, as higher oxygen pressures improve the ability of neutrophils to kill the fungi [279,280].

## 12. Conclusions and Future Perspectives

COVID-19, caused by SARS-CoV-2, has the potential to become a long-lasting global health crisis. The use of certain steroids in patients with COVID-19 compromises their immunity, and consequently, secondary infections can develop. Patients with COVID-19 have been administered a variety of steroid drugs that weaken their immune systems, and the long-term use of steroids has been found to be associated with mucormycosis or aspergillosis. Recently, the rapidly and continually developing field of nanotechnology has been widely applied in biomedical research. Nanotechnology is a viable prospective treatment, and nanomaterials based on metal ions exhibited strong efficacy against bacteria, fungi, and viruses. Typical antimicrobial agents should have a broad spectrum, high efficiency, and long-lasting antimicrobial activity. They should also be stable and have excellent biocompatibility. The use of nanoparticles as antifungal agents is possible because of their unique antimicrobial properties. As antimicrobial agents, nanoparticles have a broad antimicrobial spectrum and can inhibit the growth of fungi. In this review, we have discussed the impact of silver, zinc oxide, gold, copper, titanium, magnetic, iron, and carbon nanoparticles on various types of fungi. In general, nanoparticles inhibit fungal cell viability by allowing the binding of Ag^+^ to sulfhydryl (thiol) and phosphate groups in the cell wall of fungi, which leads to interference with the electron transport chain and the generation of energy. Furthermore, nanoparticles inhibit DNA replication and the respiratory chain in fungi, ultimately causing cell death by the generation of ROS. Although nanoparticles have significant applications in biomedicine, concrete guidelines should be devised by experts from industries, governments, and the scientific community to reduce black fungal infection by using nanoparticles. Nanoparticles are promising tools for immune modulation, either by stimulating or suppressing the immune response.

To control COVID-19-related black fungus infection, rapid diagnosis and intervention are essential. This includes controlling blood sugar levels, the urgent removal of dead tissue, and treatment with antifungal drugs. Controlling these fungal infections will require increased awareness and better tests to diagnose them early, along with a focus on controlling diabetes and using corticosteroids appropriately. Patients require access to timely surgery and antifungal treatment. However, further research is also required to learn more about how to prevent such infections. Because of its rapid progression, angioinvasive nature, the swift diagnosis of mucormycosis is essential, and treatment should be initiated as quickly as possible after diagnosis. Early diagnosis, tight glycemic control, and elimination of steroid use are recommended. Prompt radical surgical debridement, liposomal amphotericin B, and posaconazole are the current standard of care. The role of dietary nutraceuticals can be evaluated as a supplemental measure. To overcome the rapid increase of infections caused by the delta variant, quicker diagnoses, better treatment methods, and more research are needed [281].

CAM is an emerging global threat and a significant challenge during the COVID-19 pandemic. CAM is governed by environmental, host, and iatrogenic factors. The involvement of all these factors needs to be evaluated to determine the reason for the unprecedented surge in CAM cases globally. The molecular mechanism between uncontrolled diabetes and CAM needs to be studied extensively. It will also be necessary to determine the pathogenic potential of Mucorales due to SARS-CoV-2 infection. 

Nanoparticles are playing a significant role in extensive antifungal effects; however, several factors need to be considered for biomedical applications including harmful effects, bioavailability, cellular interactions, bio-distribution, and biodegradation level of NPs. The accumulation of NPs in the environment and human body and uptake by biological systems leads to catastrophic consequences such as DNA and membrane damage, protein misfolding, and mitochondrial damage. Hence, clear investigations are necessary for widening their applications and successful commercialization. Further studies are required to understand the development of the unique properties of these mystic particles.

## Figures and Tables

**Table 1:** Antifungal effects of various types of nanoparticles.

S. No.	Type of Nanoparticles	Size	Target Fungi	References
		(nm)		
1	AgNPs	17.0	*A. niger, A. flavus,* and *F. oxysporum*	[193]
2	AgNPs	23.0	*V. dahliae*	[194]
3	AgNPs	10–50	*A. alternata, S. sclerotiorum,* *M. phaseolina, R. solani,* *B. cinerea, and C. lunata*	[195]
4	AgNPs	50	*A. flavus and P. chrysogenum*	[196]
5	AgNPs	20	*B. maydis*	[197]
6	AgNPs	29	*R. nigricans*	[198]
7	AgNPs	10–20		
			*Colletotrichum* sp., *F. oxysporum, F. acuminatum,* *F. tricinctum, F. graminearum,* *F. incarnatum, R. solani,* *S. sclerotiorum,* and *A. alternate*	[199]
8	AgNPs	75		[200]
9	AgNPs	50	*A. alternata and C. lunata.*	
				[201]
			*H. rostratum, F. solani,* *F. oxysporum,* and *A. alternate*	
10	AgNPs	10–20		[202]
			*A. flavus and A. parasiticus*	
11	AgNPs	5–30		
			*F. oxysporum, F. moniliform,* *F. solani, F. verticillioides,* *F. semitectum, A. flavus,* *A. terreus, A. niger, A. ficuum,* *P. citrinum, P. islandicum,* *P. chrysogenum, R. stolonifer,* *Phoma, A. alternata, and* *A. chlamydospora*	[203]
12	AgNPs	16–20	*A. alternate, A. niger,* *A. nidulans, C. herbarum,* *F. moniliforme, Fusarium* spp.,	[204]
13	AgNPs	25–40	*F. oxysporum*, and *T. harzianum*	[205]
14	AgNPs	10	*A. flavus, A. niger, A. tereus,* *P. notatum, R. olina, F. solani,* *F. oxysporum, T. viride,* *V. dahlia,* and *P. spinosum*	[206]
15	AgNPs	15–400	*Alternaria* sp., *F. oxysporum,* *F. moniliforme,* and *F. tricinctum.*	[207]
		40–90	*B. cinerea, P. expansum, A. niger,* *Alternaria* sp., and *Rhizopus* sp.	[208]
16	CuNPs			
		20–40		
17	CuNPs			
			*A. flavus*, *A. fumigates*, and *F.* *oxysporum.*	
		200–500		[202]
18	CuNPs		*A. flavus* and *A. parasiticus*	
		10		[136]
19	CuNPs		*F. solani, Neofusicoccum* sp.,	
		3–60	and *F. oxysporum.*	
				[209]
20	CuNPs			
		30–300		
			*A. niger, A terreus*, and	[210]
21	CuNPs		*A. fumigatus*	
		22		
			*Alternaria* spp., *A. niger,*	[211]
			*Pythium* spp., and	
22	ZnONPs	10–25	*Fusarium* spp.	
			*A. alternata, A solani,*	
			*F. expansum*, and	[212]
23	ZnONPs	10–25	*Penicilliun* sp.	
			*B. albicans, C. glabrata, C. tropicalis*	[213]
24	TiO2 NPs			
		70–300		
			*Candida sps*	[213]
25	SeNPs			
			*Candida sps*	
				[214]
			*Candida albicans*	

**Table 2:** Microorganisms involved in the pathogenesis of COVID-19.

S. No.	Place of Organ	Name of Microorganisms	Reference
1	Oral	*Streptococcus, Porphyromonas, Abiotrophia, Enterobacter, Neisseria mucosa, Veillonella parvula,* *Lactobacillus fermentum, Enterococcus faecalis, Atopobium parvulum, Acinetobacter baumannii,* *Prevotella melaninogenica, jejuni, denticola,* and *oris; Eikenella corrodens; Capnocytophaga sputigena* *and gingivalis;* and *Aggregatibacter aphrophilus), Aspergillus* sp., *Nakaseomyces* sp., and *Malassezia* sp., *Candida* sp., *Saccharomyces* sp., *Epstein–Barr virus, Staphylococcus phage ROSA, Streptococcus* *phage EJ-1, phage PH10, Lactobacillus phage phiadh*.	[259]
2	Lung	*Burkholderiacepacia complex (BCC), Staphylococcus epidermidis, Mycoplasma* spp. (including *M. hominis* and *M. orale)*	[260]
		*Cutaneotricosporon, Issatchenkia, Wallemia, Cladosporium, Alternaria, Dipodascus, Mortierella,* *Aspergillus, Naganishia, Diutina,* and *Candida*	[261]
3	Lung	*Lung alphaherpesvirus 1, rhinovirus B, human orthopneumovirus*	[262]
4	Lung	*Acinetobacter, Chryseobacterium, Burkholderia, Brevundimonas, Sphingobium, Enterobacteriaceae*	[261]
5	Gut	*Streptococcus, Rothia, Actinomyces, Vellionella, Collinsella aerofaciens, Collinsella tanakaei, Streptococcus infantis, Morganella morganii, Candida albicans, Candida auris, Aspergillus flavus, Coprobacillus* sp., *Clostridium ramosum, Clostridium hathewayi*	[263,264,265]
